# Genetic Mapping of Loci for Resistance to Stem Rust in a Tetraploid Wheat Collection

**DOI:** 10.3390/ijms19123907

**Published:** 2018-12-06

**Authors:** Antonietta Saccomanno, Oadi Matny, Daniela Marone, Giovanni Laidò, Giuseppe Petruzzino, Elisabetta Mazzucotelli, Francesca Desiderio, Antonio Blanco, Agata Gadaleta, Nicola Pecchioni, Pasquale De Vita, Brian Steffenson, Anna Maria Mastrangelo

**Affiliations:** 1 Council for Agricultural Research and Economics, Research Centre for Cereal and Industrial Crops, 71122 Foggia (FG), Italy; antoniettasaccomanno@yahoo.it (A.S.); daniela.marone@crea.gov.it (D.M.); giovanni.lai79@libero.it (G.L.); pinospazio@gmail.com (G.P.); nicola.pecchioni@crea.gov.it (N.P.); pasquale.devita@crea.gov.it (P.D.V.); 2 Department of Plant Pathology, University of Minnesota, Saint Paul, MN 55108, USA; onmatny@umn.edu (O.M.); bsteffen@umn.edu (B.S.); 3 Council for Agricultural Research and Economics, Research Centre for Genomics and Bioinfomatics, 29017 Fiorenzuola d’Arda (PC), Italy; elisabetta.mazzucotelli@crea.gov.it (E.M.); francesca.desiderio@crea.gov.it (F.D.); 4 Department of Agricultural & Environmental Science, Research Unit of “Genetics and Plant Biotechnology”, University of Bari, 70126 Bari, Italy; blanco@agr.uniba.it (A.B.); agata.gadaleta@uniba.it (A.G.); 5 Council for Agricultural Research and Economics, Research Centre for Cereal and Industrial Crops, 24126 Bergamo (BG), Italy

**Keywords:** tetraploid wheat, stem rust, resistant loci

## Abstract

Stem rust, caused by *Puccinia graminis* f. sp. *tritici* (*Pgt*), is a major biotic constraint to wheat production worldwide. Disease resistant cultivars are a sustainable means for the efficient control of this disease. To identify quantitative trait loci (QTLs) conferring resistance to stem rust at the seedling stage, an association mapping panel consisting of 230 tetraploid wheat accessions were evaluated for reaction to five *Pgt* races under greenhouse conditions. A high level of phenotypic variation was observed in the panel in response to all of the races, allowing for genome-wide association mapping of resistance QTLs in wild, landrace, and cultivated tetraploid wheats. Twenty-two resistance QTLs were identified, which were characterized by at least two marker-trait associations. Most of the identified resistance loci were coincident with previously identified rust resistance genes/QTLs; however, six regions detected on chromosomes 1B, 5A, 5B, 6B, and 7B may be novel. Availability of the reference genome sequence of wild emmer wheat accession Zavitan facilitated the search for candidate resistance genes in the regions where QTLs were identified, and many of them were annotated as NOD (nucleotide binding oligomerization domain)-like receptor (NLR) genes or genes related to broad spectrum resistance.

## 1. Introduction

Durum wheat (*Triticum turgidum* L. var. *durum*) is economically and nutritionally important for the production of couscous and alimentary pasta particularly in Mediterranean countries, which account for approximately 75% of global durum wheat production [1]. Among the most important fungal diseases of durum wheat are the three rusts (stem, leaf, and stripe) that may cause up to 50% yield loss, mainly due to a reduction in biomass, harvest index, and kernels per square meter [2]. Rust infection may also lead to downgrading of grain quality, another economic loss for producer. Stem rust, caused by *Puccinia graminis* Pers. f. sp. *tritici*, Eriks. & E. Henn, is often considered the most devastating of the three rust diseases of wheat because it can cause complete crop loss over a widespread area within a short period of time [3,4,5].

Resistant cultivars provide one of the best means for controlling stem rust. To date, nearly 60 *stem rust* (*Sr*) loci and many quantitative trait loci (QTLs) have been identified in wheat and its wild relatives against stem rust [6]. Among these, several are described as race non-specific resistance genes, including *Sr57*, *Sr58*, *Sr55*, and *Sr2* [7]. Despite the large number of *Sr* genes described, only a few are still effective in different regions of Europe and North Africa, due to the remarkable ability of the pathogen to change and overcome the deployed resistance genes. New widely virulent races of *Pgt* occasionally emerge and spread, threatening wheat production on a global scale. For example, in the United States races TPMKC and TTTTF overcame several widely used *Sr* genes [8,9]; in Uganda race TTKSK (isolate Ug99) was discovered to carry virulence for the widely deployed resistance gene *Sr31* and now variants in the lineage possess virulence for *Sr24*, *Sr36*, *Sr9h,* and *SrTmp* and have spread across eastern and southern Africa [10,11,12]; and in Ethiopia three races were described TRTTF, JRCQC and TKTTF with virulence on resistance genes *Sr9e*, *Sr13,* and *SrTmp* [13,14]. Such virulent races threaten not only the wheat-producing regions in which they were first detected, but also distant areas due to the ability of the pathogen to be disseminated across long distances region by wind [15]. For all of these reasons, it is of paramount importance to conduct studies aimed at identifying new resistance genes that can be used in durum wheat breeding programs.

Many studies have been carried out in order to identify and map genes for stem rust resistance in wheat. Tetraploid wheats (*T. turgidum* ssp.) in particular have contributed a number of important stem rust resistance genes such as *Sr2*, *Sr9d*, *Sr9e*, *Sr9g*, *Sr11*, *Sr12*, *Sr13*, *Sr14,* and *Sr17* [5,6,16,17]. Most of these studies were based on the analysis of biparental populations, but more recently, genome-wide association studies (GWAS) have been employed. This mapping approach includes panels of genotypes that are not necessarily related to each other, e.g., wild relatives, landraces, and breeding germplasm. Due to the large number of genotypes that can be evaluated with GWAS, many novel QTL can be discovered, as was demonstrated with stripe rust resistance in a common wheat panel using GWAS [18]. GWAS refers to statistically significant associations identified between molecular markers and the phenotypic trait when the marker and the causal gene are in linkage disequilibrium (LD), by means of models which are chosen depending on the population and the data under analysis. LD, defined as the non-random association of alleles at different loci, has to be carefully considered in association mapping. Patterns of LD vary depending on several factors: in general, LD is greater in self-crossing than in out-crossing species and it can vary with species, population structure and genomic region [19]. Within the same species, cultivars have much higher LD extent than landraces, which, in turn, exhibit longer LD blocks than the wild forms, as demonstrated in a panel of tetraploid wheat genotypes in which durum wheat cultivars were compared to domesticated and wild emmer accessions [20]. In general, LD can extend from 5 to 20 cM in élite durum wheat cultivars analyzed with simple-sequence repeat (SSR) or diversity arrays technology (DArT) markers [20,21,22,23], but lower LD has been observed when a large number of single nucleotide polymorphism (SNP) markers was used in the analysis [24,25].

In GWAS studies, usually markers with a position on genetic maps are used. The availability of the sequence of a genome for a crop species allows to obtain the physical position of MTAs directly on a pseudomolecule and to analyze with a great precision the chromosome region of the MTA in terms of the presence of candidate genes.

The aim of the present study was to evaluate a tetraploid wheat collection for resistance to five *Pgt* races under the controlled conditions of the greenhouse. This tetraploid wheat panel was previously evaluated for resistance to the widely virulent *Pgt* race TTKSK and significant marker-trait associations were identified with SSR and DArT markers [26]. The phenotypic data collected in response to the five *Pgt* races (TPMKC, TTTTF, JRCQC, TRTTF, and TKTTF) plus TTKSK were analyzed by a GWAS approach with nearly 16,000 SNP markers in order to dissect the genomic architecture of stem rust resistance in durum wheat.

## 2. Results

### 2.1. Evaluation of the Tetraploid Wheat Collection for Resistance to Stem Rust

Optimal infection levels were achieved in all experiments with each race, allowing for the clear scoring of ITs in the germplasm. Statistically significant differences (*p* < 0.001) were found across genotypes, but also across subspecies for all of the *Pgt* races used in the study. High heritability values were observed, ranging from 81% for race JRCQC to 92% for race TKTTF, indicating the robustness of the data and the low error rate (Table 1).

**Figure 1:**
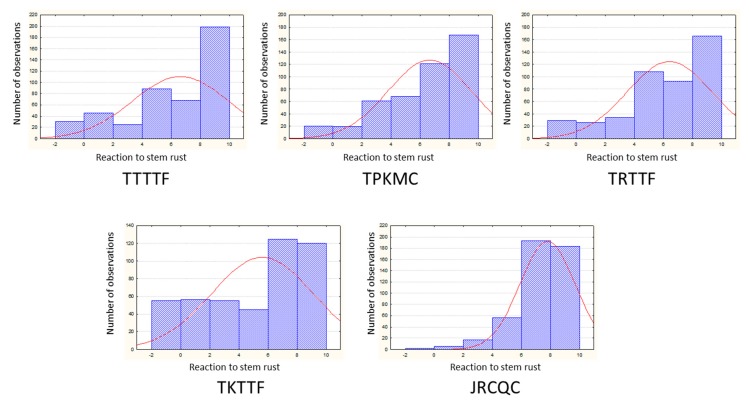
Distribution frequencies for the reactions of the whole collection to the five races of *Puccinia graminis* f. sp. *tritici*.

The frequency distribution for the reaction to infection with the five races in the whole collection suggests a complex genetic control for these traits (Figure 1). A prevalence of susceptible genotypes was observed for all of the races, especially JRCQC. The observed skewness did not affect the significance level of statistical tests.

**Figure 2:**
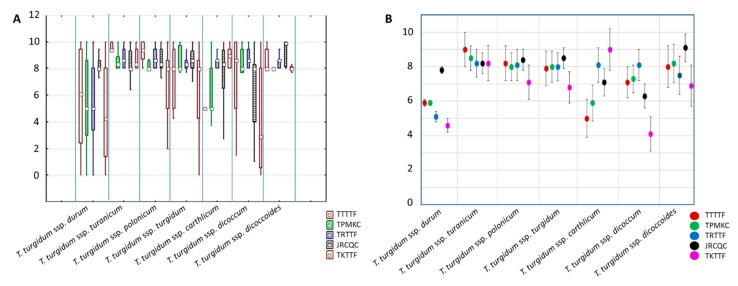
Phenotypic reaction to *Puccinia graminis* f. sp. *tritici* of the different subspecies. (**A**) Box plot showing the phenotypic variation for reactions of the tetraploid wheat subspecies groups to the five races of *Puccinia graminis* f. sp. *tritici*. (**B**) Detail of the mean values for reactions of the tetraploid wheat subspecies groups to the five races of *Puccinia graminis* f. sp. *tritici*. The vertical bars indicate the confidence interval at *p* < 0.05.

A high level of variation was observed in response to all of the five races in the tetraploid wheat collection, although some differences were noted across subspecies depending on the race (Figure 2A). As an example, the wild emmer group was characterized by very limited phenotypic variation for all of the *Pgt* races. Other groups showed wide variation in response to some races and limited variation to others, e.g., with domesticated emmer (*T. turgidum* ssp. *dicoccum*) to races TTTTF, JRCQC, and TKTTF vs. TPMKC and TRTTF. The durum subgroup was the most variable in response to all of the races except JRCQC.

The tetraploid subspecies were also different in terms of their resistance to the five *Pgt* races (Figure 2B). The durum subgroup and the *T. turgidum* ssp. *carthlicum* genotypes showed, in general, the highest level of resistance to races TTTTF and TPMKC. The durum subgroup also exhibited high levels of resistance to races TRTTF and TKTFF, as did the *T. turgidum* ssp. *dicoccum* group to the latter race. Very virulent race was JRCQC, for which high susceptibility levels were observed in all the subspecies. With respect to individual accessions within the subspecies, genotypes were identified with resistance to all of the tested *Pgt* races. These genotypes are mainly durum wheat cultivars (Altar 84, Athena, Durfort, Granizo, Grazia, L092, Rusticano, Avispa, Sansone, Svevo, Varano, Virgilio, and Baio), an accession of *T. turgidum* ssp. *polonicum* (PI 366117), and an accession of *T. turgidum* ssp. *dicoccum* (MG 5300). All of these same genotypes were also resistant to race TTKSK with the exception of Sansone, Varano, Baio, and MG 5300 [26].

### 2.2. Association Mapping

Association mapping analyses were conducted with the MLM + K model because it proved to be the most appropriate for the datasets produced in the present study (Figure S1).

In a previous study, a similar approach was used to identify the genetic determinants of resistance to TTKSK in the Ug99 lineage of African *Pgt* races [26]. In that study, significant associations were identified between the resistant phenotype at seedling stage and SSR and DArT markers. An enhanced genotyping effort of the tetraploid wheat collection with the wheat 90K SNP array [27] allowed for a more comprehensive GWAS analysis that included the five races used in this investigation as well as with race TTKSK used by [26]. Taking into account two classes of statistical significance (FDR < 0.05 and FDR < 0.1), several MTAs were identified across the six *Pgt* races (including TTKSK), but only QTL regions represented by sets of at least two closely linked markers are shown in the present study (Table S1). The marker with the highest probability was considered as the QTL-tagging marker (Table S1). Twenty-two QTL regions were identified on chromosomes 1B, 2B, 3B, 4A, 5A, 5B, 6A, 6B, and 7B. Some of the identified QTL regions were of particular interest as they were involved in the reaction to two races. The QTL region 9, mapped to chromosome 4A, was found associated with resistance to races TRTTF and TTTTF in the Q2 dataset. In both cases, the association was highly significant (FDR < 0.05), and the percentage of explained variation was 20% or higher for both races. Other QTL regions involved in resistance to two races were the 14 (chromosome 6A, races JRCQC and TRTTF) and 20 (chromosome 7B, races TRTTF and TPMKC). Both regions were identified in the Q2 dataset, and for the second one the R^2^ range was up to 22.4%. Another interesting QTL was in region 13 on chromosome 5B. In this case the region was found associated with resistance to two races in two different subsets: the whole collection for the race TTKSK, and the Q2 group for the race JRCQC.

For some QTL regions, a highly significant association was identified with two races and both the associations were confirmed in two different datasets. Such was the case with QTL region 10 on chromosome 4A in response to races JRCQC and TKTTF, which was confirmed in the whole collection and in the durum subset. The R^2^ range was 7.8% in the whole collection to 28.4% in the durum subgroup for race JRCQC and 9.4% to 23.5% for the respective subgroups to race TKTTF.

The wheat tetraploid panel was also evaluated for adult resistance to a composite of stem rust races in the field. The adult plant assessment for many of the tetraploid wheat accessions could not be obtained because they did not reach the heading stage, probably due to their photoperiod sensitivity. Although the experimental design included just a single replicate for each genotype, thereby precluding a statistically valid evaluation of the resistance QTLs, the analyses nonetheless confirmed two QTL regions identified at the seedling stage: n. 5 on chromosome 2B, and n. 21 on chromosome 7B. The QTL region 5 was found in the whole collection and in the durum subsample, while region 21 was observed in the Q2 group. The percentage of explained phenotypic variation ranged between 6.4% and 36.8%.

Most of the resistance QTLs identified in the present study were coincident with QTL previously published, providing a validation of the approach used in the present study (Table S1). Moreover, some QTLs were of particular interest because they may represent novel resistance loci based on the latest published information (see Discussion).

### 2.3. Search for Disease-Related Genes in the Chromosome Regions Corresponding to QTLs for Stem Rust Resistance

In order to identify candidate genes for resistance to stem rust, we inspected the putative function of gene sequences corresponding to the SNPs associated with the resistant phenotype (threshold E value: 10^−5^). On a total of 259 SNP markers located in the QTL regions reported in Table S1, for 11 markers a correspondence was found with disease-related genes. IWB57134 marker, within QTL region 2 on chromosome 1B corresponds to an ABC transporter, as well as three markers located in QTL region 5 on chromosome 2B (IWA8195, IWA6050, and IWB41975) and a single marker in QTL region 19 on chromosome 7B (IWB3243). QTL region 5 also contained two markers corresponding to disease resistance proteins, and one marker corresponding to *RGA2* (*Rho-type GTPase-activating protein*) gene. Other correspondences with disease resistance proteins were found for markers in QTL regions 10 and 11 on chromosome 4A (IWB34733 and IWB41334), and 16 on chromosome 6A (IWB69393).

The search for candidate genes was extended to the genes present in the confidence interval of some QTLs of particular interest. The sequence of SNP markers included in the QTL intervals were projected onto the reference genome sequence of wild emmer wheat (accession Zavitan—[28]), and the functional annotation of the genes included within the corresponding interval of the physical map was inspected. Table 2 shows the results of this search for some QTL regions of particular interest based on the R^2^ values and effect on more than one race. The confidence interval of the QTLs corresponded to regions comprised between 1.6 and 17 Mbp. The number of annotated genes retrieved in these regions were from 21 for QTL region 22 to 753 for QTL region 10. The number of annotated genes was not strictly related to the interval in Mbp, indeed the ratio between the number of annotated genes and interval size was highest for QTL region 16 with 61.4 genes per Mbp.

A clear functional annotation was available for many of these genes, so it was possible to identify those related with a reaction to pathogens for most of QTL regions taken into consideration. Disease-related genes were considered those genes annotated as very similar to known genes for resistance in several plant species, or genes corresponding to gene families known to have a role in plant immunity, as NLR genes. Very interesting were regions characterized by a high number of disease-related genes, probably organized in gene clusters. One hundred and thirty-seven disease-related genes were identified in the QTL region 10, and 48 in the QTL region 16, with a ratio of 0.18 and 0.19 disease-related genes per number of annotated genes, respectively. Some genes related to a general response to pathogen attack were present, such as those annotated for callose synthase, which were observed in QTL regions 10, 16, and 18. Nonetheless, most of the disease-related genes were resistance genes belonging to the NLR gene family. Genes similar to *RGA2* were found in QTL regions 10, 11, and 16, those similar to *RPM1* in QTL regions 10, 11, 16, 18, and 19, and those similar to *RPP13* (*Recognition of Peronospora Parasitica 13*) in QTL regions 10, 11, 13, 16, 17, and 18). Finally, 4 *MLO-like* (*Mildew resistance locus-like*) genes were identified in QTL region 10, and a *Pm3-like* gene in QTL region 22.

## 3. Discussion

Fungal diseases, and stem rust in particular, are among the most destructive biotic constrains for durum wheat production world-wide. A better understanding of the genetic basis underlying the response to different *Pgt* races is the first step to improve and enhance the disease resistance of this important crop. Several studies carried out in both the field and controlled greenhouse conditions have indicated that stem rust resistance is likely under oligenic or polygenic additive control, resulting from the cumulative effect of beneficial alleles from multiple loci (major and minor) of variable effect [26,29,30]. Thus, association mapping is widely used to identify disease resistance genes/QTLs in many crops [1,26,29,31].

In the present investigation, we report a genome-wide association study to identify chromosome regions involved in resistance to six races of *Pgt* in a structured panel of tetraploid wheat accessions (230 inbred lines), including a large set of durum wheat varieties and a representative sample of *T. turgidum* evolutionary lineages, including wild and domesticated accessions. The analysis was conducted on three datasets (whole collection, durum subsample, and Q2 group) with different LD levels and structures, as previously described [20,26]. Among the six races, JRCQC was particularly virulent in the tetraploid panel evaluated, as a smaller fraction of resistant genotypes was found compared to the other races. Similar results in proportion were reported also by [1].

Resistance sources were identified among domesticated accessions, but also within the gene pool of cultivated durum wheat. Altar 84, Athena, Durfort, Granizo, Grazia, L092, Rusticano, Avispa, Svevo, and Virgilio are durum wheat cultivars/advanced breeding lines showing a strong resistant phenotype to all of the *Pgt* races which were tested in the present study. Having effective sources of resistance in elite cultivars represents a great advantage in breeding as the resistance determinants are already present in an adapted genetic background with respect to agronomic and quality traits. In the alternative case, the transfer of a resistance locus from a wild or domesticated accession runs the risk of introducing linked deleterious alleles (i.e., linkage drag) in the recurrent parent. Nevertheless, a large number of effective resistance genes are needed by breeders to counter the evolution of virulence determinants in the pathogen. An accession of *T. turgidum* ssp. *polonicum* (PI 366117) was also of interest for its resistance to all six *Pgt* races. Further studies are needed to elucidate the genetic basis of the resistance in these genotypes. Crossing them to a completely susceptible genotype (the durum wheat cultivar Vendetta is an example from our panel) represents a first step in the strategy to dissect this trait.

**Figure 3:**
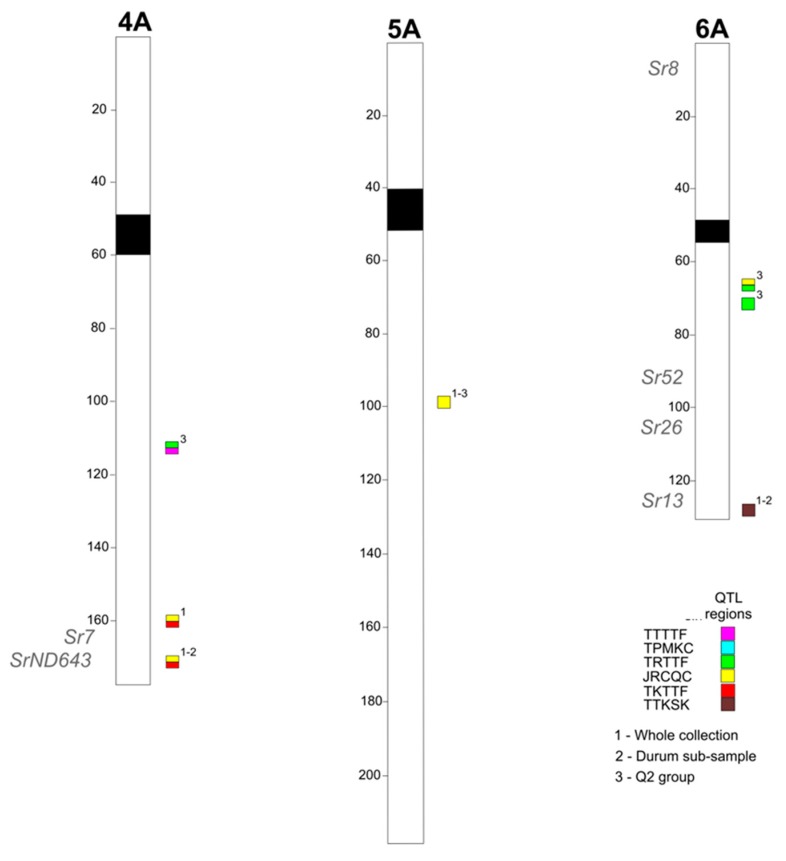

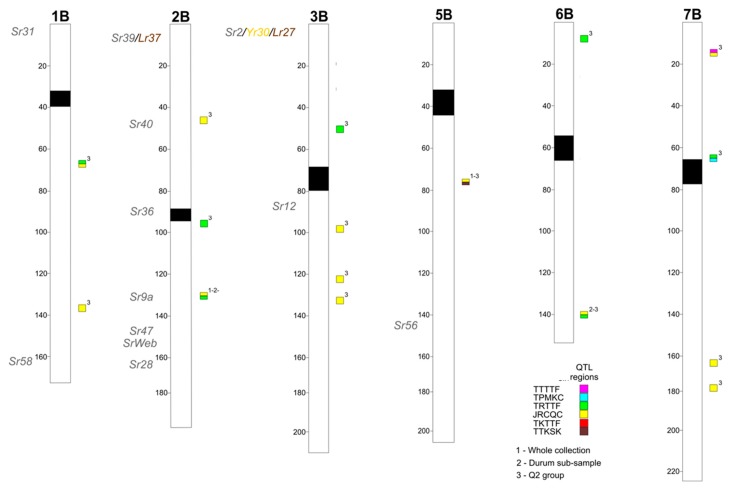
Schematic representation of a genome chromosomes of the durum consensus linkage map (Maccaferri et al. 2015) with map positions of QTLs for stem rust resistance. QTL regions were indicated on the right side and cM distances on the left side of the bar. QTLs are represented by squares on the right of each chromosome bar and a number related to a specific dataset, while the *Sr* genes on the left side.

The structured panel of tetraploid wheat accessions evaluated in the present study encompasses a large portion of the genetic variation present in the tetraploid wheat gene pool [32] and therefore is a good resource for identifying new stem rust resistance genes. To compare our results with those already published, the QTLs were projected onto the tetraploid consensus map [33]. A number of resistance QTLs identified in the present study are coincident with chromosomal positions of previously reported *Sr* genes/QTLs based on both linkage and association mapping studies performed on tetraploid and hexaploid wheats. As an example, QTL region 16 on chromosome 6A, was previously identified in biparental populations by [34], and more recently in association mapping panel of durum wheat cultivars by [1,26]. For QTL region 10 on chromosome 4A (Table S1), loci for stem rust resistance were previously identified in both durum and bread wheat, and against several stem rust races, such as TTKSK [26,35,36], TKTTF [37], and TTTTF [38]. Two *Sr* genes were also mapped to this region: *Sr7* and *SrND643* [36] (Figure 3). This region was also involved in the resistance to two races in the present study (JRCQC and TKTTF): therefore, it could contain different race-specific resistance genes/alleles, or loci conferring broad resistance. This locus, if unique in this chromosome region, could have originated very early during wheat evolution, as it has been identified in both bread and durum wheat. Another example is the QTL region 5, on chromosome 2B, which was mapped in the present study in proximity of the *Sr9a* gene [39]. The coincidence of many QTL regions identified in the present study with those already reported in the literature represents a good validation of the association mapping approach herein used.

In this study, we identified six resistance loci that appeared to map in novel chromosomal regions to the best of our knowledge. Among these novel loci are several of particular importance because they explain a large percentage of the phenotypic variation for rust reaction. As an example, QTL region 1 on chromosome 1B, identified in the Q2 group, explained up to 30% of the observed phenotypic variation. Other examples are QTL region 19, on chromosome 7B, which showed an R^2^ of 35.6% for resistance to race TTTTF, and QTL regions 8 (chromosome 3B) and 21 (chromosome 7B) explaining up to 25% and 36.8% of the observed variation for reaction to race JRCQC, respectively. QTL region 21 is particularly interesting because it was detected also in the field test. This QTL, together with the QTL region 5 on chromosome 2B, also identified in the field test, could represent important loci as acting as both seedling and adult plant resistance loci. Other putatively novel QTLs were identified on chromosomes 5A, 5B, and 6B. The fact that in some cases these QTLs were associated with resistance in two different panels, contributes to the robustness of the results of this association mapping analysis.

The availability of reference genome sequences represents a resource to investigate the gene content in the QTL interval and to proceed to gene cloning once the above-mentioned interval is reduced to a very small region by fine-mapping. The gene content of some of the most significant QTLs identified in the present study was investigated by projecting the sequence of the corresponding SNP markers within the QTL confidence interval to the genome of the wild emmer wheat accession Zavitan [28]. This approach demonstrated that, in some cases, especially for the QTLs identified in the Q2 group, where a higher mapping resolution is expected based on LD, intervals of a few Mbp could be retrieved (Table 2). The number of the genes residing in these intervals is very high. Moreover the size of resistance-related gene families as the NLR genes is so large [40,41] that the probability to find one of these genes in a given interval by chance is high. For these reasons we cannot identify strong candidate genes at this stage of the GWAS. Nonetheless, the presence of clusters of resistance genes in QTLs which may be novel (e.g., QTL region 18 on chromosome 6B) may guide us in the choice of the most suitable QTLs for validation and fine mapping, and the availability of tetraploid wheat genomes for advancing gene cloning efforts.

## 4. Materials and Methods

### 4.1. Plant Material and Pgt Races

Phenotypic evaluations were carried out on a germplasm panel consisting of 230 inbred lines classified into seven subspecies: ssp. *durum* (128), ssp. *turanicum* (20), ssp. *turgidum* (19), ssp. *polonicum* (20), ssp. *carthlicum* (12), ssp. *dicoccum* (19) and ssp. *dicoccoides* (12). Of the 128 durum wheat accessions, 96 are representative of Italian durum wheat breeding programs over the last 100 years, including 7 landraces or old durum wheat varieties (Cappelli, Aziziah, Russello, Timilia, Tangaron, Capeiti-8, and Grifoni). Laidò et al. [32] provided a detailed list of the genotypes (number/name, year of release, country, and pedigree) for each subspecies. The genetic diversity, population structure and LD patterns of this collection of tetraploid wheats are fully described in [20,32]. All tetraploid wheat accessions were evaluated for seedling resistance to races TPMKC, TTTTF, JRCQC, TRTTF, and TKTTF, under the controlled conditions of a greenhouse. Race TPMKC (74MN1409) is virulent on *Sr36* [8]. Race TTTTF (isolate 02MN84A-1-2) is the most widely virulent race reported in the United States, producing high infection types (ITs) on all but *Sr24* and *Sr31* in the 20 line wheat stem rust differential set [10,42]. Races TRTTF and JRCQC are from Ethiopia and are virulent on both resistance genes *Sr9e* and *Sr13* [13]. Race TKTTF (13ETH18-1), also from Ethiopia, is virulent on *SrTmp* [14]. Susceptible controls were included in each experiment to monitor the infection level (density of uredinia on leaves) and virulence (maximum uredinial size) of the pathogen races. Wheat cultivars McNair 701 (Cltr 15288) and Line E were the susceptible controls for the stem rust evaluation. Additionally, the respective wheat differential lines also were included in the experiments to confirm the identity and purity of races of *P*. *graminis* f. sp. *tritici* [10,42]. Resistant lines within the respective differential wheat sets served as the resistant controls.

### 4.2. Phenotypic Evaluation and Statistical Analysis

Urediniospores previously increased on susceptible wheat lines, were collected, desiccated, and stored at −80 °C until used for this study.

The stem rust evaluations with foreign *Pgt* races were conducted in the Biosafety Level-3 Containment Facility on the St. Paul campus of the University of Minnesota (USA) during the winter of 2015–2016. Six seeds of each genotype and controls were sown into plastic pots (7.6 by 7.6 by 10.8 cm [length × width × height]) filled with a 50:50 mix of steam-sterilized native soil and sunshine MVP growth medium (Sun Gro Horticulture, Quincy, MI, USA) [43]. After planting, all pots were watered and fertilized with Osmocote controlled-release fertilizer 14–14–14 (Scott’s Company, Marysville, OH, USA) (1.4 g/pot) and moved to a cold room at 4 °C for 3 days to break possible dormancy in wild type accessions. Plants were also fertilized at the primary leaf stage with Peters Dark Weather formulation 15–0–15 (Scott’s Company) (≈40 g/L at 1/16 dilution). Plants were grown in the greenhouse at 19 to 22 °C with a 14- to 16-h photoperiod supplemented by 400-W high-pressure sodium lamps emitting a minimum of 300 µmol photons m^−2^s^–1^. Twelve-day-old seedlings (first leaf fully expanded) were inoculated with urediniospores of *Pgt* races suspended in 700 µL of a lightweight mineral oil carrier (Soltrol 170; Phillips Petroleum, Bartlesville, OK, USA). Urediniospores, taken from storage in the −80 °C freezer, were heat-shocked in a water bath at 45 °C for 15 min and then placed for 2 h in a humidifier jar (RH 98%–99%, solution of KOH) before being used as inoculum. The concentration of inoculum used was 14 mg/0.7 mL oil applied at a rate of approximately 0.013 mg per plant [44]. The oil carrier was allowed to evaporate before plants were moved into the mist chambers. Plants were then misted continuously for 30 min with ultrasonic humidifiers to establish an initial layer of moisture on the surfaces; after that the humidifiers were set to run for 2 min every 15 min for 16 h in dark at 20–22 °C. Then, lights were turned on with the misters still running for 2 additional hours before the chamber doors were opened to allow slow drying of the plants. Following this infection period, plants were returned to the greenhouse under the same environmental conditions. ITs were scored on plants 12–14 days post-inoculation using the 0–4 scale described by [45], where IT = 0 represents a completely incompatible (highly resistant) reaction and IT = 4 represents a fully compatible (highly susceptible) reaction. To meet the data format required for association mapping analysis, the raw seedling IT data were converted to a 0–9 linear disease scale as follows: 0, 1−, 1, 1+, 2−, 2, 2+, 3−, 3 and 3+ were coded as 0, 1, 2, 3, 4, 5, 6, 7, 8 and 9, respectively. For lines with heterogeneous reactions, only the most prevalent IT was used. The semi-colon symbol used to represent a hypersensitive fleck ‘‘;’’ was converted to 0. IT 4 was converted to 9 [46].

All the experiments were conducted in a completely randomized design and were repeated once over time. Any accessions exhibiting variable reactions across the replicates were repeated again in a third test.

The panel was also evaluated for adult plant resistance to a bulk of U.S. races (QFCSC, QTHJC, MCCFC, RCRSC, RKQQC, and TPMKC) in the field at St. Paul, MN in 2016. Field disease ratings were based on the percentage of stem and leaf sheath tissue infected by rust using the modified Cobb scale [47]. These ratings were made on plants at the mid- to hard-dough stage of development.

The data were analyzed using an ANOVA test, the homogeneity of phenotypic variance between replications was verified. Mean disease scores for genotypes were tested for statistical differences using Fischer’s protected least significant difference at *p* < 0.05. Genetic variance (σ^2^G) and broad-sense heritability (H) for the disease phenotype data were also estimated. All data were statistically analyzed using a statistical software package (Statistica, Statsoft Inc., Tulsa, OK, USA).

### 4.3. Genotyping

Each accession, previously genotyped with 26 simple sequence repeat (SSR) and 970 Diversity Arrays Technology (DArT) markers, was further genotyped with the Illumina^®^ iSelect 90K wheat SNP assay [27] at TraitGenetics GmbH, Gatersleben, DE. The dataset filtering was carried out based on the following criteria: (1) markers showing residual heterozygosity were entered as missing values; (2) markers with less than 10% missing data and accessions with less than 20% missing data were retained; and (3) markers with minor allele frequency (MAF) greater than 10% were retained. Among these markers, those for which the genetic position was known based on a high-density consensus map of tetraploid wheat [33] were used in this study. All of the genotypic analyses were carried out on three distinct datasets as described in [20,26]: the whole collection (230 genotypes), the durum subsample (127 genotypes) and the Q2 group (98 genotypes), which mainly contains wild and domesticated accessions of the tetraploid panel. Following the above described filtering criteria, 17,678, 12,225 and 19,191 polymorphic SNP markers were retained for the association mapping analysis for the whole collection, the durum subsample and the Q2 group, respectively.

### 4.4. Association Mapping Analysis

The tetraploid association mapping panel was previously characterized in terms of population structure and linkage disequilibrium based on SSR and DArT markers [20,32]. These analyses were run again with SNP markers and a detailed description of the results will be object of a separate publication. Briefly, for the GWAS study, population structure was determined with a set of non-correlated SNP markers identified with the software Haploview [48] based on a LD threshold of 0.8. Based on this selection, 6455, 2611 and 7617 SNP markers were used for the whole collection, the durum subsample and the Q2 subgroup, respectively. The population structure was analyzed with a Bayesan model-based clustering approach by processing the molecular SNP data with the STRUCTURE software package v.2.3.4 [49] to investigate presence of genetic sub-groups. The number of sub-groups (K) was estimated by 20 independent runs for each K (from 2 to 20) applying the admixture model, with allele frequencies for the SNP markers, 100,000 Markov Chain Monte Carlo (MCMC) repetitions, and a 100,000 burn-in period. The means of the likelihood estimates for each K were calculated. The true K was determined using both an estimate of the posterior probability of the data for a given K (as proposed by [49]) and the Evanno ΔK [50] as determined with STRUCTURE HARVEST tool [51]. A genotype was considered to belong to a group if its membership coefficient was ≥0.5 [52]. Structures selected for the GWAS analysis were K = 3 for the whole collection, K = 6 for the durum subgroup, and K = 5 for the Q2 subgroup.

The LD analyses were conducted on the whole collection, on the durum sub-sample and on the Q2 group using the SNP markers mapped on the high-density consensus wheat map reported by [33] using Tassel software 5.2.18 to calculate the genome-wide LD (allele frequency correlation, r^2^). A critical value of r^2^ was identified according to [53] by root transforming the r^2^ values and taking the 95% percentile as the threshold, which explains why LD is probably connected to a real physical linkage. The intra-chromosomal r^2^ values were plotted against the genetic distance, and a line was marked using second-degree locally weighted polynomial regression (LOESS) according to [54], using an R package (http://www.r-project.org). Intercepts of the LOESS curve to the critical r^2^, were obtained to determine how rapidly LD decay occurs (Figure S2). The map distance at which LD falls below the r^2^ threshold 0.3 was used to define the confidence intervals of QTL detected in this study. This is a frequently used LD threshold for QTL detection [18,55].

TASSEL software, version 5.2.39, was used to carry out mixed linear model (MLM) analyses for association mapping. Two different models were tested in order to choose one that fit the data better: (1) MLM + Q + K, in which the Q matrix, obtained from the analysis of population structure, and the kinship matrix were integrated as covariates to correct for the effects of population substructure; and (2) MLM + K, in which only the correction for kinship was taken into account. The trimmed marker datasets based on LD (*r*^2^ = 0.8) were used to generate a marker similarity matrix containing all of the lines (K matrix) with the TASSEL software. TASSEL calculates the kinship as the proportion of alleles shared between each pair of lines. Once this matrix was calculated, the numbers were rescaled to lie between 0 and 2 [56]. The critical P values for assessment of significance of the marker trait associations (MTAs) were calculated based on a false discovery rate (FDR) of 0.05 or 0.1 [57], which is defined as the expected proportions of the true null hypotheses that are rejected. The algorithm described by [58] was used, because it controls the false discovery rate (FDR) for independent test statistics, but also for some types of positive dependence [59].

All of the MTAs mapped on the durum wheat consensus map within a short map interval (10 cM or less) and in LD with each other (r^2^ ≥ 0.3) were grouped into a single QTL [33]. To compare our results with those obtained in other studies, we considered the most recently published information on genes and QTL mapping for stem rust resistance. QTLs and MTAs previously published were compared with those identified in the present study using as a common framework the wheat tetraploid consensus map [33].

A search for candidate genes was carried out for the most significant QTLs. For each MTA, the tagging marker as well as the confidence interval were located on the consensus map according to LD threshold 0.3, that is 1 cM (whole collection and the Q2 group) or 3 cM (durum subgroup) (Figure S2). From the consensus map, the left and right markers, together with internal markers, corresponding to each confidence interval were projected to the genome assembly of the *T. dicoccoides* accession Zavitan [28] based on the matches obtained using the nucleotide sequence of SNPs [27] as queries in a BLAST search against the Zavitan genome (BLASTN, E-value = E-10). SNPs corresponding to candidate genes have been identified, moreover all of the genes contained in the corresponding intervals have been retrieved with their functional annotation.

## 5. Conclusions

In conclusion, the present study provides a collection of loci for stem rust resistance in a tetraploid wheat panel. The use of the 90K SNPs allowed us to compare our results with those of similar studies, and provided a source of molecular markers which can be useful in durum wheat breeding programs to simultaneously select multiple beneficial alleles for disease resistance [1]. Ultimately, the knowledge of the genome sequence in a species can lead to the identification of high-quality haplotypes necessary to accurately associate molecular markers with phenotypes, as demonstrated in a recent study in which more than 500 rice accessions have been re-sequenced, allowing the construction of a high-density haplotype map of the rice genome to uncover the genetic basis of 14 agronomic traits [60]. The identification, in the present and previously published studies, of many loci for resistance with small effects suggests that new approaches like genomic selection could be effective for improving stem rust resistance in durum wheat [61]. Nonetheless, the identification in the present study of some novel QTLs with large effect indicates that it could be useful to combine marker assisted selection and genomic selection into the same breeding program to fix both major-and minor-effect loci and to obtain improved lines for resistance to stem rust.

## Figures and Tables

**Table 1:** Statistic parameters for reaction of a tetraploid wheat collection to five races of *Puccinia graminis* f. sp. *tritici*. CV: coefficient of variation; MSD: minimum significant difference; H^2^: heritability.

Race	CV	Mean	Range	Min–Max	Genetic Variance	MSD	H^2^ (%)
TTTTF	0.48	6.65	10	0–10	10.34	2.05	90
TPMKC	0.41	6.69	10	0–10	7.86	1.92	89
TRTTF	0.44	6.43	10	0–10	8.16	1.73	91
JRCQC	0.23	7.82	10	0–10	3.28	1.62	81
TKTTF	0.60	5.64	10	0–10	11.70	1.95	92

**Table 2:** Size and gene content of the physical regions corresponding to some of the quantitative trait loci (QTLs) identified in the present study.

QTL Region	Interval Zavitan (Mbp)	Number of Annotated Genes	Ratio Annotated Genes/Interval Size	Number of Disease-Related Genes	Ratio Disease-Related/Annotated Genes	Ratio Disease-Related Genes/Mbp
10	17	753	44.3	137	0.18	8.06
11	3.9	62	15.9	13	0.21	3.33
13	9.8	123	12.6	8	0.07	0.82
14	9.5	80	8.4	1	0.01	0.11
16	4.2	258	61.4	48	0.19	11.43
17	2.1	34	16.2	1	0.03	0.48
18	4.8	157	32.7	12	0.08	2.50
19	5.1	70	13.7	2	0.03	0.39
20	17	77	4.5	2	0.03	0.12
22	1.6	21	13.1	4	0.19	2.50

## Supplementary Materials

Supplementary Materials can be found at http://www.mdpi.com/1422-0067/19/12/3907/s1.
