# Supplementary material for: Genetic Mapping of Loci for Resistance to Stem Rust in a Tetraploid Wheat Collection
Source: Int J Mol Sci. 2018 Dec 6;19(12):3907. doi: 10.3390/ijms19123907 (PMC6321032; doi:10.3390/ijms19123907)
Supplement: Supplementary file 1 [file ijms-19-03907-s001.zip › ijms-394491-supplementary/ijms-394491-supplementary-excel.pdf]

Table S1. QTL regions represented by MTAs identified with MLM+K model for seedling resistance to stem rust in the whole collection, in the durum sub-sample and in the Q2 group.
Probability values corresponding to FDR < 0.05 are reported in bold, whereas the values corresponding to FDR < 0.1 are written in italics. candidate genes are reported as gene accession number in the Zavitan genome and the corresponding functional annotation for each SNP marker.

Chrom.	N. QTL region	Race	Tagging Marker	Position (cM)	Whole Collection				Durum Subsample				Q2				Candidate genes	Reference
					P value	Other MTAs in the region	Range R2 (%)	Chr. Interval (cM)	P value	Other MTAs in the region	Range R2 (%)	Chr. Interval (cM)	P value	Other MTAs in the region	Range R2 (%)	Chr. Interval (cM)		
1B	1	TRTTF	IWB13329	65.2									**8.91E-08**	0	27.3	0		
		JRCQC	IWB42604	68.1									**1.20E-08**	0	29.9	0		
	2	JRCQC	IWB71824	110.6									**2.49E-10**	6	24.3-4.4	2.9	IWB5718: TRIDC1AG059570 (ABC transporter C family member 2)	[1]
2B	3	JRCQC	IWB578	45.7									**4.47E-05**	4	11.4-16.5	7.3		[1]
	4	TRTTF	IWA5927	95.2									**1.32E-07**	8	17.3-26.3	5.7		[2]
		JRCQC	IWB4750	137.9									**5.13E-07**	1	15.1-24.3	17.8		
	5	TRTTF	IWB55526	137.9	**2.61E-09**	49	6.4-16.7	14.3									IWA8195 and IWA6050: TRIDC2BG070950, and IWB41975: TRIDC2BG068750 (ABC transporter C family member 2); IWB1801 and IWB7433: TRIDC2BG071010 (Disease resistance protein), and IWB55526: TRIDC2BG071070 (Disease resistance protein RGA2).	[3-8]
		TRTTF	IWB37190	137.9					**1.04E-08**	97	8.6-25	17.7						
3B	6	TRTTF	IWB728	51.9									*7.92E-05*	4	14.8-18	0		[4,9]
	7	JRCQC	IWA2400	123									**2.06E-06**	3	17.8-21.8	3.8		[5]
	8	JRCQC	IWB4535	130.5									**2.54E-07**	3	23-25	0		[10]
4A	9	TRTTF	IWB28717	110.7									**9.02E-08**	1	25.8-27	0.2		[5]
		TTTTF	IWB8853	113.6									**6.00E-06**	0	20	0		
	10	JRCQC	IWB8733	162.8					**1.90E-10**	20	10.3-28.4	15.1						
		TKTTF	IWB13101	159.5					**1.20E-08**	44	9.4-23.5	15.1						
		JRCQC	IWA4651	162.4	*1.94E-05*	1	7.8-9.4	0.4									IWB8733: TRIDC4AG068020 (Disease resistance protein)	[1,5,8,11-16]
		TKTTF	IWB8733	162.8	**2.14E-06**	0	9.7	0										
	11	TKTTF	IWB6810	173.2					**8.88E-06**	26	10.2-18.1	6.7						
		JRCQC	IWB72930	173.6					**2.28E-07**	5	10.7-22	6.7					IWB4138: TRIDC4AG071870 (Disease resistance protein)	[5,7,8]
		JRCQC	IWB29282	173.7	*5.85E-06*	2	9-10.4	0.1										
5A	12	JRCQC	IWA6949	99	*3.92E-05*	0	7.3	0					**2.71E-05**	1	14.7-17.5	1.6		
5B	13	JRCQC	IWB40681	74.8									**5.70E-05**	1	17.4	0		
		TTKSK	IWA6468	75.4	**1.09E-07**	0	13.9	0										
6A	14	JRCQC	IWB717	66.3									**2.30E-05**	1	15.4-18.5	0.6		[17]
		TRTTF	IWA7052	66.3									*9.15E-05*	1	15.5	0		
	15	TRTTF	IWB56339	72.4									**7.50E-07**	2	19.4-23.3	0		[17]
	16	TTKSK	IWB29696	126.7					**1.82E-07**	3	19.9-24.2	3.3					IWB69393: TRIDC6AG060750 (Disease resistance protein)	[1,12,18]
		TTKSK	IWB24237	130	**4.65E-06**	2	10.1-11.2	7.1										
6B	17	TRTTF	IWB56605	7.2									*1.37E-04*	2	12-17.7	0		[4]
	18	JRCQC	IWA1484	140.1					**2.05E-04**	0	13.8	0						
		TRTTF	IWB2746	140.1									**1.01E-06**	0	23.2	0		
7B	19	TTTTF	IWB564	13.8									**4.88E-08**	0	4.6	0	IWB5718: TRIDC7BG002510 (ABC transporter C family member 2)	
		JRCQC	IWB3243	15.2									*3.26E-04*	0	13.4	0		
	20	TRTTF	IWB23791	66.2									*7.32E-05*	1	14.2-15.8	0.1		[8]
		TPMKC	IWB56619	67.9									**1.56E-06**	0	22.4	0		
	21	JRCQC	IWB38104	164.4									**6.89E-11**	4	17.6-10.8	5.7		
	22	JRCQC	IWB8577	179.6									**1.28E-05**	4	13.9-18.8	2.2		[14,19]
