# Supplementary material for: Genetic Mapping of Loci for Resistance to Stem Rust in a Tetraploid Wheat Collection
Source: Int J Mol Sci. 2018 Dec 6;19(12):3907. doi: 10.3390/ijms19123907 (PMC6321032; doi:10.3390/ijms19123907)
Supplement: Supplementary file 1 [file ijms-19-03907-s001.zip › ijms-394491-supplementary/ijms-394491-supplementary_figures.pdf]

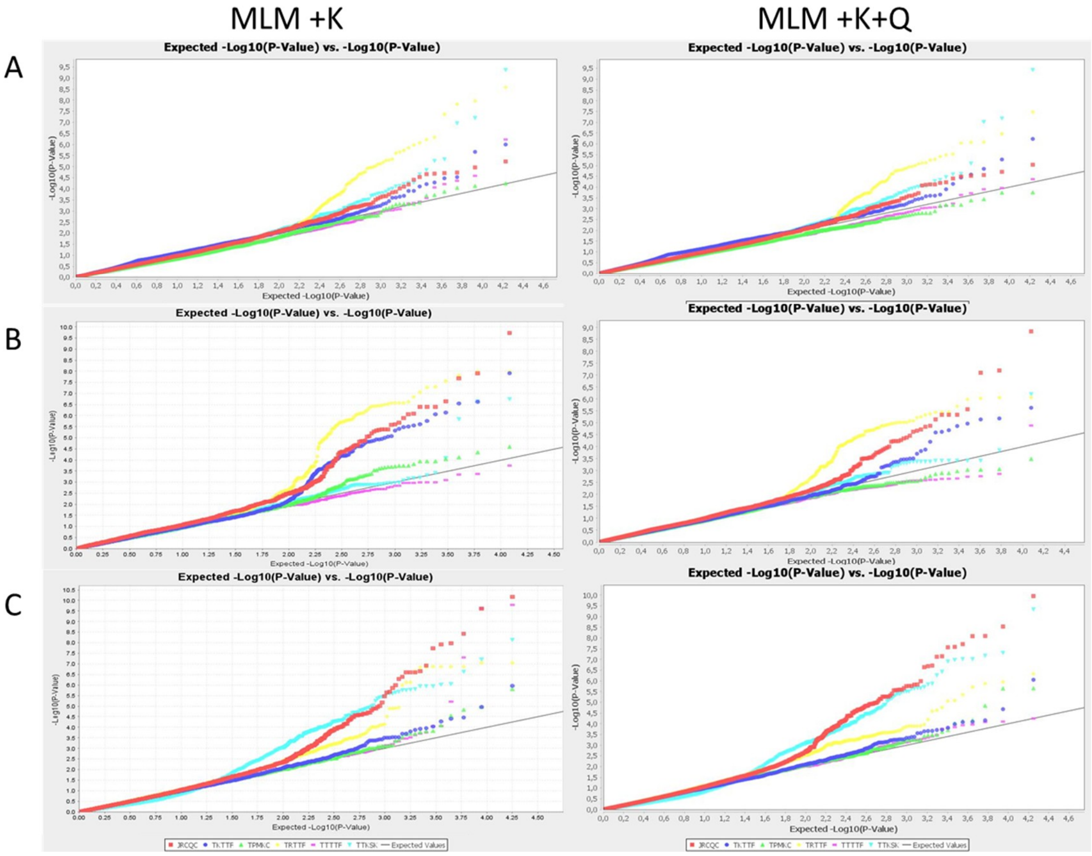

Figure S1. QQ-plot determined by MLM+K and MLM+K+Q statistical models for stem rust seedling experiments (6 races) for A: Whole collection, B: Durum sub-sample, C: Q2 .

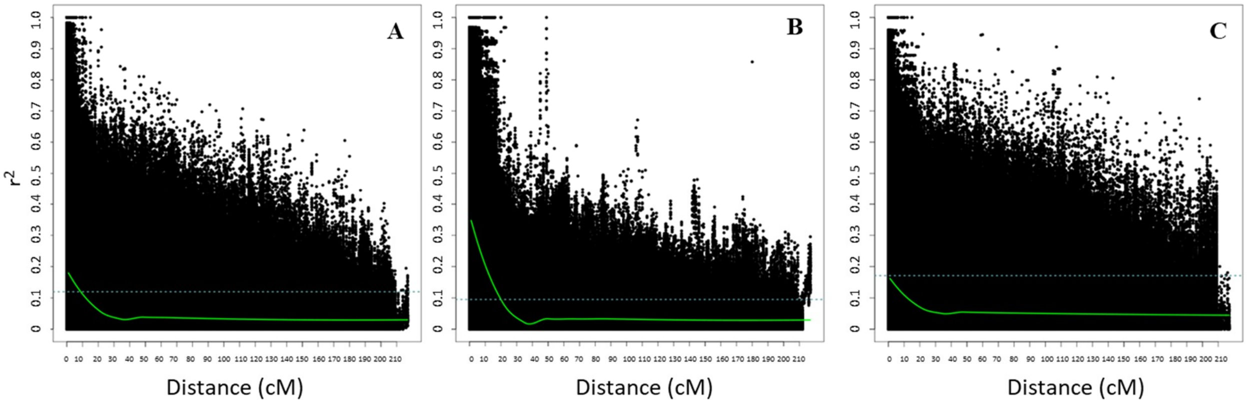

Figure S2. Overview of the LD parameter r^2 for the whole genome of the intrachromosomal pairs in the whole collection (A), in the durum sub-sample (B), and in Q2 group (C). The scatterplots show the distributions of the LD parameter r^2 according to the genetic distance. The horizontal line indicates the 95% percentile of the distribution of the unlinked r^2, which gives the critical value of r^2.
